# The Efficacy of Anti-Tumor Necrosis Factor Therapy in Cryopyrin-Associated Periodic Syndromes: A Report of Two Cases

**DOI:** 10.1155/2022/2898553

**Published:** 2022-03-03

**Authors:** Fatemeh Tahghighi, Mahdieh Vahedi, Nima Parvaneh, Mohammad Shahrooei, Vahid Ziaee

**Affiliations:** ^1^Department of Pediatrics, Tehran University of Medical Sciences, Tehran, Iran; ^2^Children's Medical Center, Pediatrics Center of Excellence, Tehran, Iran; ^3^Pediatric Rheumatology Iranian Society, Tehran, Iran; ^4^Department of Pediatrics, Mashhad University of Medical Sciences, Mashhad, Iran; ^5^Department of Microbiology and Immunology, Laboratory of Clinical Bacteriology and Mycology, KU Leuven, Leuven, Belgium; ^6^Pediatric Rheumatology Research Group, Rheumatology Research Center, Tehran University of Medical Sciences, Tehran, Iran

## Abstract

**Background:**

Cryopyrin-associated periodic syndromes (CAPSs) are a group of autoinflammatory disorders caused by a mutation in the NLRP3 gene. NLRP3 mutations increase inflammasome activation; therefore, IL-1 targeted therapies are frequently used in the aforementioned disorders. *Case Presentation*. We report two cases of CAPS in which the diagnosis was confirmed by genetic tests and an evaluation of the therapeutic response to anti-tumor necrosis factor (anti-TNF) agents.

**Conclusion:**

IL-1 inhibitors are highly effective in treating CAPS patients. Most patients with severe symptoms such as neurologic involvement improve with IL-1 blockade. Anti-TNF agents might be effective in reducing mild manifestation; however, they are not effective in improving more severe complications.

## 1. Introduction

Cryopyrin-associated periodic syndromes (CAPS) are a group of autoinflammatory disorders caused by a gain-of-function mutation in the NLRP3 (CIAS1) gene located on the long arm of chromosome 1. CAPS are a spectrum of disorders with a range of severity. The moderate form of the CAPS is Muckle–Wells syndrome, often inherited as an autosomal dominant trait. The main clinical manifestations include recurrent episodes of fever, urticaria-like rash, and ocular and articular involvement. The severe form of the CAPS is the chronic infantile neurological cutaneous and articular (CINCA) disease that is presented with urticaria-like rash, mental delay, arthritis, and sensorineural hearing loss [1–5].

Formation of NLRP3 inflammasome leads to caspase 1-mediated release of the proinflammatory cytokines such as IL-1B and IL-18. Since NLRP3 mutations can result in increased inflammasome activation and IL-1 production, IL-1 targeted therapies are used frequently in the patients. Interleukin 1 receptor antagonists such as anakinra can improve CAPS symptoms and prevent major complications such as hearing loss and amyloidosis [6–9]. Some patients with CAPS show partial response to IL-1 blockade. Other biologics such as anti-tumor necrosis factor (anti-TNF) agents are less effective than IL-1 inhibitors. In a few patients, anti-TNF agents have been able to result in partial response and improvement. TNF-*α* may play a role in regulating and activating the NLRP3 inflammasome [1, 2, 10].

We will describe two cases of CAPS and evaluate the responses to anti-TNF agents and IL-1 inhibitors. The current study aims to describe the relative effectiveness of anti-TNF agents in improving CAPS symptoms, highlighting the role of TNF-*α* in the pathogenesis of the disorders.

## 2. Case 1

**Figure 1:**
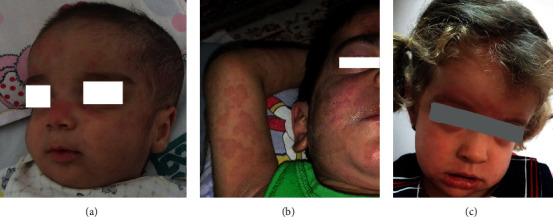
Urticaria-like rash on face and extremities. (c) At 10 months old, (b) at 3 years old (the first case), and (a) at 2 years old (the second case).

A 1-year-old boy was brought to our clinic with joint pain and swelling in his left ankle and knees that had lasted for more than 14 days. Since birth, he had had a recurrent urticarial rash that was not triggered by cold or other physical stimuli (Figure 1). Often presented with high-grade fever, the rash lasted 24 hours and resolved without scarring. Additionally, he was treated with antihistamines for a long time due to a history of chronic urticaria. He was born premature and admitted for 60 days in the neonatal intensive care unit (NICU). His parents were not blood relatives. His older brother has a history of chronic atopic dermatitis. On examination, neurodevelopmental evaluation result was normal, but his weight and height were below the 3rd percentile for his age. He had significant synovial hypertrophy in both knees. No hepatosplenomegaly or lymphadenopathy was present. Other physical examinations indicated normal results. However, laboratory findings showed leukocytosis, anemia, and thrombocytosis. Moreover, there was an increase in acute phase reactants. The patient's laboratory tests are listed in Table 1. Both chest X-ray and abdominal ultrasonography were normal. The left ankle and right knee sonography showed mild to moderate effusion and synovial hypertrophy.

**Figure 2:**
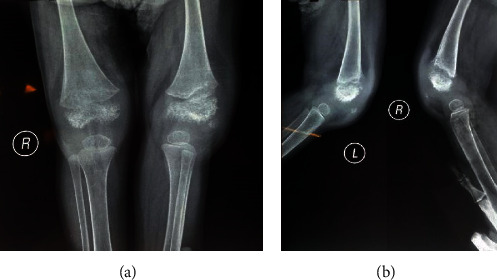
Knees X-ray shows metaphyseal irregularity, widening of growth palate, and soft tissue swelling (the first case).

**Figure 3:**
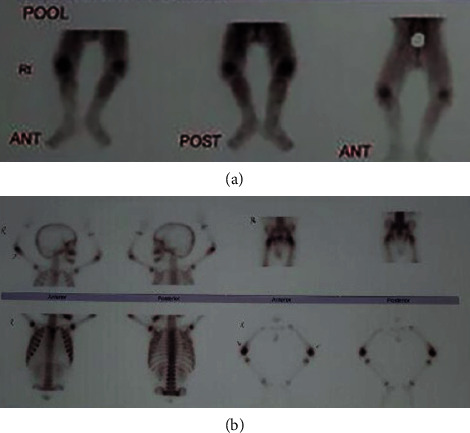
Three-phase bone scintigraphy shows inflammatory processes in several joints, including both left and right knees. (a) Immediate phase and (b) delayed phase (for the first case).

**Figure 4:**
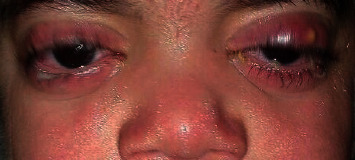
Swelling and erythema of both eyelids (the first case).

Echocardiography expressed normal ventricular function and no pericardial effusion. He had normal bone marrow aspiration and biopsy. Due to prolonged fever, arthritis, and an increase in acute phase reactants, the diagnosis of systemic juvenile idiopathic arthritis was established for him. Therefore, he was treated with prednisolone, methotrexate, and naproxen. At the age of three, he was readmitted to the hospital because of his inability to walk due to arthritis, persistent fever, and urticarial rash. An X-ray of the knees indicated metaphyseal irregularity, widening of growth palate, and soft tissue swelling (Figure 2). In three-phase bone scintigraphy, there were inflammatory processes in several joints, including the right elbow and both knees (Figure 3). At that time, the diagnosis of autoinflammatory disorders, especially CAPS, was made for him. A genetic test was performed, and the whole exome sequencing (WES) identified a new variant in exon 5 of NLRP3 (NM-001079821: c. G1060T, p. A354S) (9). The patient was heterozygous for this mutation, while his parents were homozygous wild type. It was a de novo mutation. The patient was treated with etanercept at a dose of 0.8 mg/kg per week subcutaneously because anakinra was not available at the time. After six months of anti-TNF therapy, joint involvement relatively improved. Not only could he walk but also his fever was under control. However, he had a mild effusion in his left knee during treatment with etanercept. Furthermore, the urticarial-like rash had decreased but did not resolve completely. At the age of 4, he developed both eyelids' swelling and erythema, which decreased his visual acuity (Figure 4). Ophthalmologic examination showed retinal vasculitis and severe optic disc edema. When anakinra was available, treatment was initiated. Anakinra was initiated at a dose of 1 mg/kg subcutaneously daily. After one month, a considerable response was observed, while full recovery occurred after four months. We reported this case in detail in our previous report [11].

## 3. Case 2

A 2-year-old girl was referred to our clinic for periodic fever and rash. Fever often worsened at night; besides, she had abdominal pain during some febrile episodes. She had had a recurrent purpuric urticarial rash since three months (Figure 1). The rash was usually accompanied by the onset of fever. The immunological evaluation result was normal, and she had been treated with antihistamines due to a history of chronic urticarial rash. She did not have a history of seizures, joint involvement, or conjunctivitis. She was the third child of the family. Her parents were blood relatives, and her siblings were healthy. She was hospitalized at the age of 1, with a diagnosis of sepsis and at the age of 3, for an evaluation of fever with unknown origin. Physical examinations revealed a high-grade fever, urticarial rash, and subcutaneous nodule in the subcostal and inguinal region. No hepatosplenomegaly or lymphadenopathy was present. There were no conjunctivitis, arthritis, or mental and physical disability. Neurologic examination results were normal. However, laboratory findings reported leukocytosis, anemia, and thrombocytosis. Erythrocyte sedimentation (ESR) and C-reactive protein (CRP) levels were elevated. The patient's laboratory tests are listed in Table 1. Chest X-ray and echocardiography were normal. Three-phase bone scintigraphy did not report clear evidence of inflammatory or active bone lesion. Skin biopsy showed dermis vessels with plump endothelial cells, intramural and perivascular neutrophils, and few eosinophils with no evidence of granuloma or necrosis. Also, she had normal bone marrow aspiration and biopsy. She had been treated with prednisolone and ibuprofen due to a diagnosis of systemic juvenile idiopathic arthritis; however, fevers and skin rashes had not been taken under control. The autoinflammatory disease was suggested as a diagnosis. Whole exome sequencing reported a variant in exon 3 NLRP3 gene (NM_001127462: c. G1057C, p. V353L). She was heterozygous for this variant. It was a de novo mutation. She was treated with etanercept at a dose of 0.8 mg/kg per week subcutaneously. Thereafter, fever was relatively controlled, but the rash did not respond to anti-TNF therapy. Relapse occurred after four months of treatment with etanercept. Anakinra was initiated at a dose of 1 mg/kg subcutaneously daily, and a dramatic response was observed after two months.

The signs and symptoms of the two patients are compared in Table 2.

## 4. Discussion

It is important to know that autoinflammatory syndromes are rare; however, they should be considered in any patient with recurrent or persistent inflammation. Most patients with these disorders experience delays in diagnosis. Our first patient had a fever, urticarial rash, and arthritis. He had been treated with systemic juvenile idiopathic arthritis (SJIA) diagnosis, but he was diagnosed with CAPS after further evaluation. The second patient had a recurrent fever and urticarial rash, but there was no joint involvement. She was initially treated with a diagnosis of SJIA, but her final diagnosis was Muckle–Wells syndrome after more evaluation and genetic tests.

The symptoms of cryopyrin-associated periodic fever syndromes are variable presented with a broad range of clinical manifestations [2]. Inheritance of CAPS is usually autosomal dominant. Additionally, the disease has a spectrum of symptoms in different generations. The severity of symptoms increases as the age goes up. For example, the disease may be seen as amyloidosis and renal failure in the first generations. However, in younger children, urticaria, and fever may be observed. CNS manifestations are one of the common clinical presentations of CINCA. In Muckle–Wells syndrome, joint involvement is mild to moderate, while severe joint involvement and deformity are more common in CINCA syndrome. Our first case had severe joint disease correlated with CINCA syndrome, but CNS manifestations and mental disability were not observed. Our first case also had optic disc edema that is usually presented in CINCA syndrome [11–15].

In young children with Muckle–Wells, hearing loss and amyloidosis does not usually occur, and the only manifestation of the disease may be recurrent urticarial rash and fever. The second case had typical symptoms of Muckle–Wells, including urticaria-like rash and fever. Nevertheless, she did not have joint involvement. The primary treatment for CAPS is the IL-1 inhibitors, which can completely relieve the disease's symptoms [10, 16, 17].

Both of our patients were initially treated with anti-TNF agents because IL-1 inhibitors were unavailable. In the first case, joint involvement and fever were resolved with anti-TNF agents; yet, the urticarial rash did not respond to this therapy, and optic disc edema occurred during the anti-TNF therapy. The second case was initially treated with an anti-TNF agent (etanercept) too. Nevertheless, the response was transient, and relapse occurred after four months. Some of the CAPS manifestations, such as fever, were resolved with anti-TNF agents, but some of them did not resolve fully, and relapse might occur. After IL-1 inhibitors therapy, all of the symptoms resolved dramatically and entirely.

TNF-*α* might play a regulatory role upstream of the NLRP3 inflammasome. Caspase 11-mediated inflammasome activation participates in driving the production of TNF-*α*. However, the cellular source of the TNF-*α* and the mechanism of generation are unclear. In patients with CAPS, proinflammatory cytokines such as IL-1B play an essential role in the development of symptoms. At least half of the patients with CAPS have a range of neurological manifestations at some course of illness. A few studies have shown that IL-1 inhibitors are effective for treating neurological symptoms and preventing severe CNS complications. The most common neurologic manifestations in CAPS are headache, aseptic meningitis, seizures, papilledema, and hearing loss. Serious neurological complications include optic atrophy and mental disability. Skin rash, fever, and arthritis are milder CAPS symptoms [17–20].

## 5. Limitations

In case reports, causal inference is not plausible. Response to the treatment could be a mere coincidence. Accordingly, the effects of anti-TNF agents on clinical presentations from the current case report of CAPS cannot be generalized.

## 6. Conclusion

Based on our experiences in this study, although TNF-*α* inhibitors effectively reduced mild manifestations such as fever and rash, they were not effective for improving more serious complications like papilledema and arthritis. Since CAPS is a rare disease, there are few reports of this disorder. Future studies are needed to evaluate the effect of such medical treatments on improving the various symptoms of CAPS.

## Figures and Tables

**Table 1:** Laboratory tests of cryopyrin-associated periodic syndromes cases.

LAB (unit)	Case 1	Case 2
WBC (*µ*l)	16100	16000
Hemoglobin (g/dl)	7.5	9.5
HCT (%)	25.4	35.5
Platelets (*µ*l)	567000	703000
GRA (%)	52	45
LYM (%)	40	35
MON (%)	3.9	14.1
EOS (%)	4	4.9
BASO (%)	0.1	1
ESR (mm/hr)	60	112
CRP (mg/l)	75	180
AST (U/l)	17	29
ALT (U/l)	11	30
LDH (U/l)	347	450
Uric acid (mg/dl)	3.3	4
Ferritin (ng/ml)	29	167
BUN	35	40
Creatinine	0.4	0.5
ANA	N	N
CANCA	N	N
P-ANCA	N	N
ACE	34	—
RF	N	N
ACPA	N	—
IgG (g/l)	1667	1740
IgM (g/l)	122	160
IgA (g/l)	181	178
IgE (g/l)	1021	1210
IL6 (IU/l)	28	50

LAB: laboratory; WBC: white blood cell count; HCT: hematocrit test; GRA: granulocytes; LYM: lymphocytes; MON: monocytes; EOS: eosinophils; BASO: basophils; ESR: erythrocyte sedimentation rate; CRP: C-reactive protein; AST: aspartate aminotransferase; ALT: alanine aminotransferase; LDH: lactate dehydrogenase; BUN: blood urea nitrogen; ANA: antinuclear antibody; C-ANCA: antineutrophil cytoplasmic autoantibody; P-ANCA: perinuclear antineutrophil cytoplasmic antibodies; ACE: angiotensin-converting enzyme; RF: rheumatoid factor; ACPC: anti-citrullinated peptide antibody; Ig: immunoglobulin; IL: interleukin; N: negative.

**Table 2:** Signs and symptoms of cryopyrin-associated periodic syndromes cases.

Sign and symptoms	Case 1	Case 2
Urticarial rash	+	+
Conjunctivitis	−	−
Dysmorphic features	−	−
Joint involvement	+	−
Hearing loss	−	−
Visual loss	+	−
Aseptic meningitis	−	−
Optic disc edema	+	−
Intellectual disability	−	−

## Data Availability

The data used to support the ﬁndings of this study are available from the corresponding author upon request.
